# Clothoid: An Integrated Hierarchical Framework for Autonomous Driving in a Dynamic Urban Environment

**DOI:** 10.3390/s20185053

**Published:** 2020-09-05

**Authors:** Saba Arshad, Muhammad Sualeh, Dohyeong Kim, Dinh Van Nam, Gon-Woo Kim

**Affiliations:** Intelligent Robots Laboratory, Department of Control and Robot Engineering, Chungbuk National University, Cheongju-si 28644, Korea; sabarshad1000@gmail.com (S.A.); er.sualeh@gmail.com (M.S.); robotdevel@naver.com (D.K.); quangnam.auto.tech@gmail.com (D.V.N.)

**Keywords:** autonomous vehicle, HD map, localization, perception, path planning, V2X

## Abstract

In recent years, research and development of autonomous driving technology have gained much interest. Many autonomous driving frameworks have been developed in the past. However, building a safely operating fully functional autonomous driving framework is still a challenge. Several accidents have been occurred with autonomous vehicles, including Tesla and Volvo XC90, resulting in serious personal injuries and death. One of the major reasons is the increase in urbanization and mobility demands. The autonomous vehicle is expected to increase road safety while reducing road accidents that occur due to human errors. The accurate sensing of the environment and safe driving under various scenarios must be ensured to achieve the highest level of autonomy. This research presents Clothoid, a unified framework for fully autonomous vehicles, that integrates the modules of HD mapping, localization, environmental perception, path planning, and control while considering the safety, comfort, and scalability in the real traffic environment. The proposed framework enables obstacle avoidance, pedestrian safety, object detection, road blockage avoidance, path planning for single-lane and multi-lane routes, and safe driving of vehicles throughout the journey. The performance of each module has been validated in K-City under multiple scenarios where Clothoid has been driven safely from the starting point to the goal point. The vehicle was one of the top five to successfully finish the autonomous vehicle challenge (AVC) in the Hyundai AVC.

## 1. Introduction

At the highest level of autonomy, the desired goal is to develop a fully autonomous vehicle that can drive in the dynamic urban environment. Many researchers have focused their efforts in this area. Since 2002, the Defense Advanced Research Projects Agency (DARPA) has been organizing autonomous driving challenges to support the development of fully autonomous driving technology for self-driving cars [1]. DARPA held the first self-driving car competition in 2004 and awarded the winners with one million dollars [1]. In 2007, DARPA organized an autonomous driving competition on busy roads, named the DARPA Urban Challenge where, vehicles have to navigate on roads and in unstructured zones. This competition was won by Boss [2], a vehicle integrated with 17 different sensors. Later, many autonomous driving challenges and tests have been organized. The Intelligent Vehicle Future Challenge was held in China from 2009 to 2013 [3]. The European Land-Robot Trial (ELROB) has been conducted since 2006 [4]. In South Korea, Hyundai has been organizing autonomous vehicle contests since 2009 to design and develop technology that fulfills the requirements of fully autonomous vehicles and to bring self-driving technology onto the actual road [5]. Similarly, automobile companies compete to embed autonomous technology in their vehicles. Some of the examples include Waymo [6], Autopilot [7], Autoware [8], Apollo [9], and NVIDIA DriveWorks [10]. Though they achieve the autonomy up to some extent, the development of a fully autonomous vehicle that can drive safely on the road with real traffic is still a challenge [11].

In 2019, Hyundai organized an autonomous vehicle contest in K-City. The major goal of this contest was to build an autonomous car, without using off-the-shelf autonomous vehicle solutions, that runs in self-driven mode on the road [5]. Compared with previous competitions, this contest necessitated that vehicles should be able to meet unique challenges, such as:

1. Maintenance of safety margins at all times.
2. Pedestrian detection on the road.
3. Detection and avoidance of static and dynamic obstacles.
4. Detection of road blockage regions along the path and search for the alternate shortest path.
5. Accurately follow the lane as per driving rules.
6. Pre-emption of the lane in the presence of emergency vehicles.
7. Drive in the GPS-denied area.

In this research, Clothoid, a robot operating system (ROS)-based [12] unified software framework for fully autonomous self-driving vehicles, is proposed, as it addresses the above-mentioned challenges. Clothoid is an integrated framework consisting of all the modules necessary for driving in a fully autonomous mode, such as HD mapping, localization, environmental perception, path planning, and control. An array of sensors is calibrated, including global positioning system (GPS), inertial measurement unit (IMU), light detection and ranging (LiDAR), and stereo camera, and they are set in place for environmental perception aided by a vehicle-to-everything (V2X) communication channel. The HD map is developed and fused with the LiDAR point cloud map to ensure accurate localization. The map fusion enables a robust localization in the GPS-denied regions. The long-range path planning is realized with the use of V2X communication with infrastructure and other entities, ensuring dynamic route planning in the given route network. The local path planner makes use of the dynamic route information efficiently for the generation of optimal local trajectories. Furthermore, the local path planner also utilizes the perceived environment information to optimize the trajectory in the presence of dynamic obstacles and emergencies. The controller performs the behavior control by handling the steering, throttle, and braking maneuvers. The proposed framework integrates the software components of each module in a hierarchical architecture which allows the rapid development and testing of the proposed framework. The framework has been tested at each module level and the whole system level. Through multiple tests, it is ensured that the framework successfully meets the required challenges.

The major contributions of this research include the following:

1. A unified software framework for fully autonomous driving in the real environment including the highway and urban area is developed.
2. The proposed framework can robustly detect and avoid static and dynamic obstacles in real-time.
3. It ensures the capability of driving in a GPS-denied environment.
4. Fused HD map with a LiDAR point cloud map for accurate localization of the vehicle.
5. A novel V2X-based global path planner enables the vehicle to change the path in severely dynamic conditions along the route.

K-City is known to be the world’s first autonomous vehicle testing site connected by a 5G network [13]. The road network consists of the highway and urban areas [14]. To evaluate the performance of the proposed framework, various tests have been conducted on the K-City proving ground. The vehicle has been tested in the K-City proving ground to ensure safe driving in the real environment.

The rest of the paper is organized as follows: Section 2 discusses the vehicle’s basic platform with the sensors, power and computational resources used to build the autonomous system and the general architecture of Clothoid. In Section 3, the sensor calibration is discussed. Section 4 explains the HD mapping, and Section 5 discusses the localization of a vehicle in open area and GPS-denied regions. In Section 6, the perceptual setup is described. The path panning consisting of global and local path planning is explained in Section 7. Section 8 presents the formation of the controller of Clothoid, followed by a conclusion in Section 9.

## 2. The Proposed Autonomous Driving Framework: Clothoid

### 2.1. Vehicle Platform

**Figure 1:**
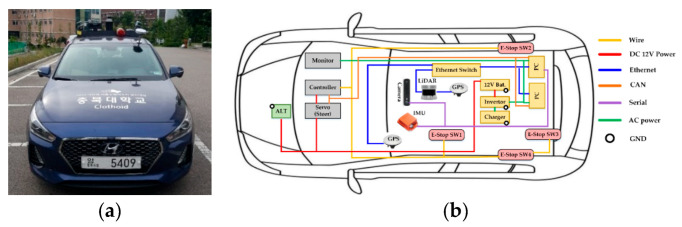
(**a**) External view of Clothoid with sensors labeled; (**b**) the Clothoid sensory and drive-by-wire system.

Clothoid is implemented on Hyundai I-30 1.6T-GDI vehicle with a modified sensory and control system, shown in Figure 1a. The vehicle platform has 2-wheel drive (2WD), and a seven-level dual clutch transmission (DCT). Throttle, brakes, and steering are actuated through the vehicle’s chassis controller area network (CAN). The DC motor attached to the steering is designed to be controlled by electrical signals. The vital vehicle information including wheel speed and steering angle is directly pulled via the CAN bus.

The vehicle platform utilizes auxiliary power generation to support sensors and computing. The auxiliary power system is a high voltage generator that is driven by the engine, installed in the bonnet of the vehicle. For safe and reliable control of the vehicle, the generator provides up to 2 kilowatts of power to the auxiliary battery depending on acceleration throttle through the use of a power control system that is managed by the power control box. The power control box contains circuit breakers to protect other sensors and circuits; a CAN bus; and a series of power converters that convert the power from the high voltage, produced by the generator, to both direct and alternating current busses needed by the electronic equipment, while constantly managing power output, voltage levels, and temperatures of the generator and converters. 

For accurate localization and perception, the vehicle is equipped with a combination of sensors that fulfills the necessary sensory requirements. The sensory information is fused to ensure the robustness in the case of failure during operation. The selected sensors are a 64-channel 3D LiDAR Ouster OS-1 [15], GPS, IMU, and a stereo ZED camera [16]. These sensors are mounted on the roof rack to achieve the highest benefits of sensor data visibility. LiDAR detects depth and intensity in 360 degrees. The camera faces the front of the vehicle with 1024 × 600 resolution and has a field of view (FOV) of 90° (H) × 60° (V) × 100° (D), limited to the front of the vehicle. The region immediately in front of the vehicle can be regarded as a blind-spot, but only for the short objects that are occluded by the vehicle bonnet. However, the visibility of the system is certainly greater than the human counterpart due to the elevation of sensors. Two global navigation satellite system (GNSS) antennas, attached on the roof, receive latitude, longitude and altitude based on L1/L2 band signals from GNSS antennas. To achieve positional accuracy, real-time kinematic (RTK) positioning was received from digital multimedia broadcasting (DMB). Figure 1b illustrates Clothoid’s sensory and hardware communication system.

An emergency stop (E-stop) switch is installed in Clothoid to ensure safe driving and avoidance of critical accident situations. It helps to immediately stop the car. E-stop can be operated through a remote controller or the switch fixed near the driving seat.

Clothoid’s main computational resources include two workstations, each running the Ubuntu 16.04 LTS operating system and consisting of Xeon X5690 eight-core 3.4 GHz processors and one NVIDIA GeForce GTX-1080Ti with 32 GB RAM and 512 GB SSD.

### 2.2. Software Architecture

**Figure 2:**
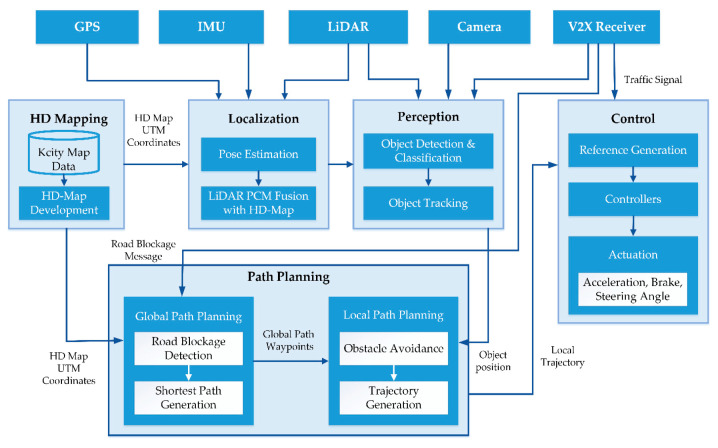
The software architecture of the proposed framework—Clothoid.

The overall architecture of Clothoid is shown in Figure 2. The major modules of the architecture include HD mapping, localization, perception, path planning, and control.

The HD mapping module reads the route network data from the database, converts the data into the Universal Transverse Mercator (UTM) coordinate system, and develops an HD map. The HD map is the key to accurate localization and navigation throughout the route.

The localization module uses the combination of sensors to accurately localize the vehicle. The localizer fuses the LiDAR point cloud map with the HD map and estimates the pose error. Due to the HD map, Clothoid can localize itself accurately with a pose error of less than 0.5 m in the outdoor environment as well as in the GPS-denied regions.

The perception component holds vital importance to the autonomous navigation of a vehicle. Clothoid’s perception module is devised to address an environmental setup that mimics a mix of rural–urban fringes and is responsible for the interpretation and fusion of information from multiple sensors for the understanding of the local environment to the rest of the system. The perception module locates the obstacles, moving vehicles, lane markings, toll gates, tunnels, and pedestrians on the road.

The path planner consists of the global path planner and the local path planner. The global path planner computes the cost, as a function of time, of all possible routes from the source to the destination with given knowledge of the route network stored in the database. It searches for an optimal path to the destination and integrates the information provided by the V2X receiver about the road congestion or blockages, construction, and the speed limit for each region along the route. Using this information, the global path is updated and is followed by the local path planner. The local path planner is responsible for the generation of the smooth trajectory at each instant by avoiding the obstacles detected by the perception module from the current position of the vehicle to the next waypoint along the generated global path.

The controlling module consists of the reference generation and the controllers. For each generated trajectory, a reference generation submodule is responsible for velocity profiling which is based on the perceived environment. The reference generation module associates the velocity with the generated trajectory and forwards it to the controllers. The actuation for acceleration, steering, and brake is performed by the longitudinal and lateral controllers based on the received trajectory.

## 3. Sensor Calibration

The GPS is calibrated to the center of the vehicle frame, and vehicle position coordinates are converted to universal transverse mercator (UTM) coordinates. For environment perception and the vehicle’s localization in GPS-denied regions, the camera and LiDAR are calibrated. The intrinsic and extrinsic parameters of camera calibrations are computed using Equation (1).

(1) $$\left[\begin{array}{c} x\\ y\\ 1 \end{array}\right]\;=\;\left[\begin{array}{ccc} f_{x} & skew\_cf_{x} & c_{x}\\ 0 & f_{y} & c_{y}\\ 0 & 0 & 1 \end{array}\right]\left[\begin{array}{ccc} r_{11} & r_{12} & r_{13}\\ r_{21} & r_{22} & r_{23}\\ r_{31} & r_{32} & r_{33} \end{array}\;\;\begin{array}{c} t_{x}\\ t_{y}\\ t_{z} \end{array}\right]\;\left[\begin{array}{c} \begin{array}{c} X\\ Y\\ Z \end{array}\\ 1 \end{array}\right]$$

**Figure 3:**
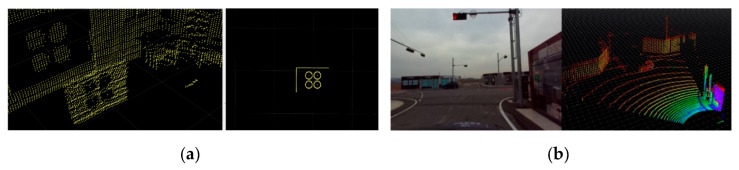
(**a**) Camera and LiDAR circle feature extraction: left (LiDAR frame), right (camera frame); (**b**) camera and LiDAR calibration: left (LiDAR points into image frame), right (point cloud).

The focal length (*f_x_, f_y_*), principal point (*c_x_, c_y_*), and skew coefficients (*skew_cf_x_*) for intrinsic parameters matrix are obtained by camera calibration with a checkerboard. The spatial location of each point in 3D space is represented with X, Y, Z coordinate axes and is projected into a 2D image plane (*x,y*) through extrinsic parameters. The extrinsic parameter matrix contains the rotation [$r_{11},\;r_{12},\;\ldots r_{33}$] and translation [$t_{x};\;t_{y};\;t_{z}]$ elements. These extrinsic parameters were obtained by using the calibration method proposed in [17]. This method extracts the circular feature points of the calibration board from the LiDAR frame and the camera frame, respectively, to obtain rotation and translation, as shown in Figure 3a. With the help of extrinsic parameters, the LiDAR 3D data are projected into an image frame, illustrated in Figure 3b, which enables *Clothoid* to detect and localize the 3D objects in the image frame.

## 4. HD Mapping

**Figure 4:**
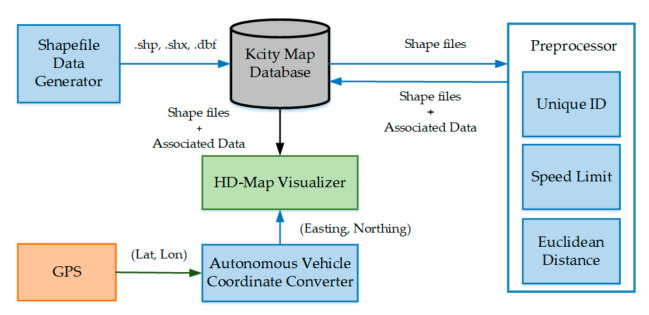
HD map development procedure.

The HD mapping module of the proposed framework generates an HD map for the autonomous vehicle. It consists of the following components: a shapefile data generator, a preprocessor, an autonomous vehicle coordinate converter, and a HD map visualizer, as shown in Figure 4.

### 4.1. Shapefile Data Generator

For the development of the HD map, firstly, the structure of the K-City ground is captured from Google maps [18] and is converted to UTM coordinates. In the UTM coordinate system, the projection along the X axis is termed “easting”, and that along the Y axis is known as “northing”. It splits the flattened Earth surface into 60 vertical planes, known as zones. These zones are sequentially numbered 1 through 60, from west to east. Each zone is 6° of longitude in width. In the vertical direction, each zone is divided into 24 latitude bands labeled with alphabetical letters A to Z, excluding “I” and “O”. The height of each of the 20 bands, C to X, is 8°, while the bands “A”, “B”, “Y” and “Z” consists of the south and north corners of the hemisphere [19,20]. 

According to the UTM coordinate system, the K-City proving ground lies in the following region:

1. Zone: 52
2. Zone vertical band: S
3. Northings: (4123586.03 — 4124647.03)
4. Eastings: (302353.88 — 302670.95)

**Figure 5:**
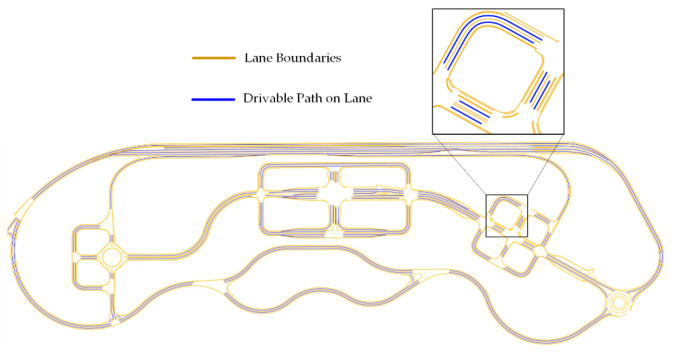
Shapefile data representation of the K-City map.

The shapefile data generator produces the shapefiles of road lanes and the drivable path between the lanes, shown in Figure 5, by using an open-source geographical information system (QGIS) [21]. These generated files contain the shapes in the spatial data format. The three mandatory files used for defining the environment structure into spatial data format include:

1. The main shapefile (.shp), which contains the geometry of features such as point, line, and area.
2. A shape index file (.shx) containing the index for each shape in the shapefile.
3. A database file (.dbf) containing the attribute information of the features.

### 4.2. Preprocessor

After generating the spatial data format, the shapefiles are preprocessed before constructing an HD map. This component reads the shapefiles of the K-City map and associates the following data to the shapes.

#### 4.2.1. Unique ID

To perform the path planning and localization of the vehicle through the HD map, each route on the map must be uniquely identifiable. For this purpose, a unique ID is assigned to each edge and node. 

#### 4.2.2. Speed Limit

The K-City map consists of an urban area and a highway area as well. The urban area consists of bus stops, a school zone, traffic light intersections, and steep curves. Thus, the maximum speed limit is not the same for both urban and highway regions. Clothoid is designed to follow the traffic rules. Thus, for each region, the map data are preprocessed to associate the speed limit according to the structure of the road.

#### 4.2.3. Euclidean Distance

In the shapefiles, each edge, which is not a straight line, is composed of multiple straight sub-edges. For each edge, the Euclidean distance computation component computes the Euclidean distance between nodes of each sub-edge and adds these distance values to obtain the total distance between the two end nodes of the edge. The total Euclidean distance value is assigned to the edge as a cost value. Later, this distance value is used by the global path planner for computation of the shortest path.

### 4.3. Autonomous Vehicle Coordinate Converter

This component constantly receives the autonomous vehicle position at 10 Hz frequency from GPS. The position information is in WGS84 coordinates [22], such as latitude and longitude. For the localization of the vehicle in the HD map, the position coordinates are converted from WGS84 to the UTM coordinate system. The resulting easting and northing coordinates are forwarded to the HD map visualizer at the same frequency.

### 4.4. HD Map Visualizer

**Figure 6:**
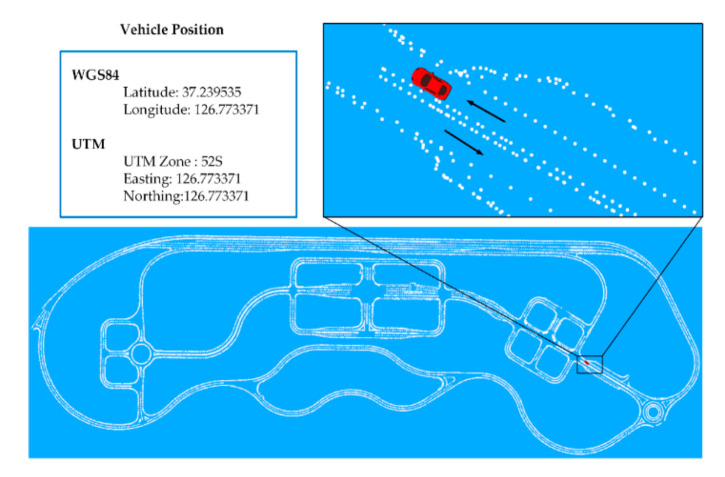
Autonomous vehicle localization on the point cloud HD map.

Visualization is performed by the use of a 3D visualization tool, RviZ [23]. The HD map visualizer reads the preprocessed shapefiles and produces the HD map using the point cloud library [24,25]. The RviZ reference frame is centered at (0,0). To keep the HD map centered with the RviZ reference frame, an offset value is subtracted from the whole map coordinates. The same offset value is subtracted from the vehicle position coordinates, received from the autonomous vehicle coordinate converter, for localization of the vehicle in the HD map. Figure 6 illustrates the HD map of K-City with the vehicle coordinates in both coordinate systems, WGS84 and UTM.

## 5. Localization

The basic sensor used for the localization is GPS, which estimates the global position based on the signals of at least four satellites. Though GPS has excellent localization performance in open grounds, it is not reliable in GPS-denied areas such as tunnels because pose error increases and a signal blocking problem occurs when satellite signals are not received directly into the rover system. To avoid the aforementioned problem, different methods have been proposed to efficiently localize the vehicle, such as camera-based lane detection [26,27], LiDAR-based point cloud map (PCM) matching [28], and IMU odometry [29]. Clothoid uses the combination of sensors i.e., GPS, IMU, and LiDAR for better estimation of vehicle pose and performs the localization by using PCM-based pose estimation and IMU odometry. Due to the high frequency of the IMU sensor, Clothoid successfully estimates the pose for short intervals. Furthermore, the LiDAR PCM matching method provides a robust vehicle pose.

The LiDAR-based localization consists of two parts: LiDAR map fusion with the HD map and unscented Kalman filter (UKF)-based localization.

### 5.1. Lidar Point Cloud Map Fusion with the HD Map

**Figure 7:**
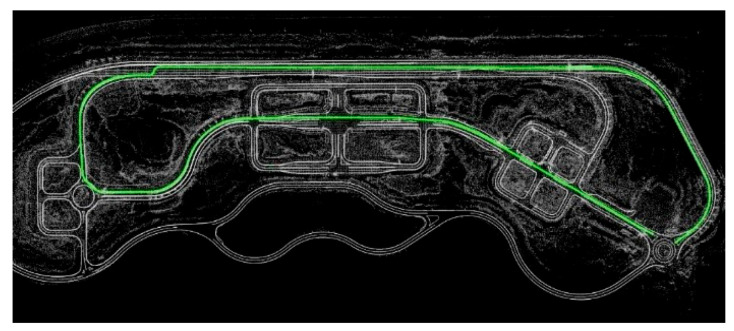
Lidar point cloud fused with the HD map.

**Figure 8:**
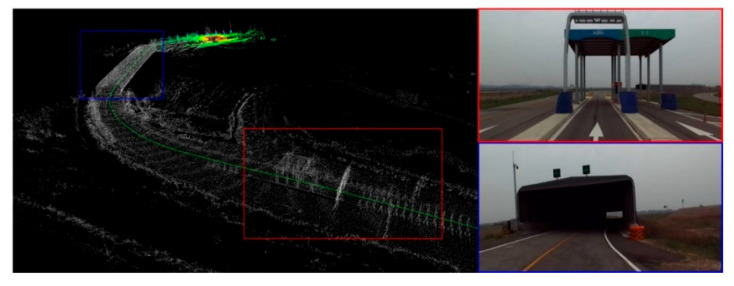
Localization through the point cloud map fused with the HD map under a tunnel and toll gate.

Firstly, the LiDAR point cloud map is fused with the K-City HD map, created by the HD mapping module, as shown in Figure 7. The point cloud map is created by using the GPS data and graph SLAM in the form of a voxel. The PCM matching error is less than 30 cm. The fused point cloud with HD map is used for the localization of a vehicle under the tunnel and toll gate, as shown in Figure 8.

### 5.2. UKF-Based Localization

As Clothoid performs localization through the use of multiple sensors, to ensure the accurate localization and reduce the pose error, the localizer implements the unscented Kalman filter [30], a variant of well-known multiple sensor-based localization method known as the Kalman filter [31]. UKF is selected because of suitability for nonlinear systems and better approximation than the extended Kalman filter [32].

In the implementation of UKF, the IMU is used for the prediction, while the correction is performed with the measurements of GPS and LiDAR, shown in Table 1. The IMU sensor is used in the prediction stage due to faster operation cycles in comparison to the other sensors. For pose estimation in the local frame using LiDAR odometry, the edge and planar features are extracted from each layer of LiDAR 3D point cloud data [33]. The LiDAR odometry measures the position in local coordinates, thus, before starting LiDAR odometry, it is necessary to take the previous position and perform position recognition in the global frame.

**Figure 9:**
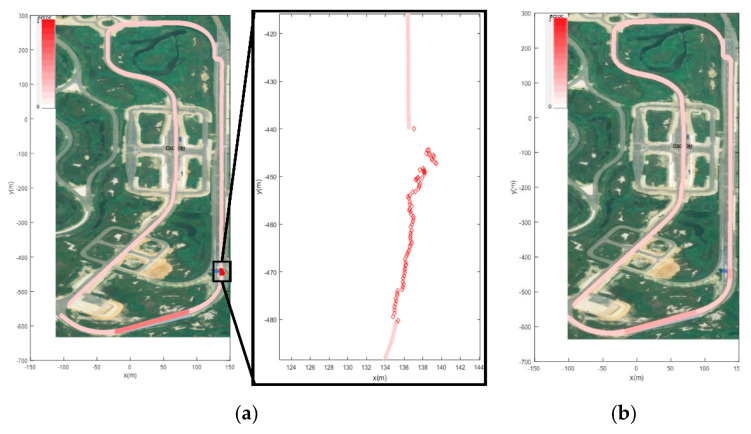
(**a**) KF localization with only GPS coordinates. Color intensity represents the pose error in outdoor and GPS-denied region i.e., the tunnel and toll gate. (**b**) Improved localization in GPS-denied regions.

**Figure 10:**
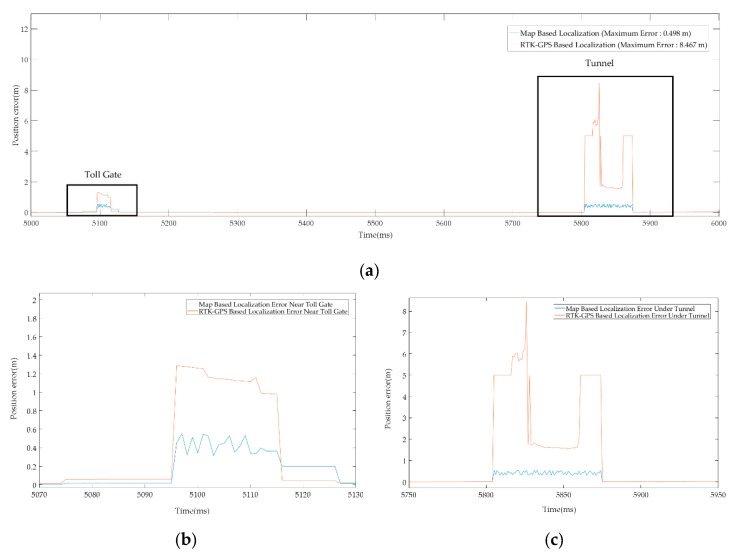
(**a**) Localization error recorded along compete trajectory. (**b**) Localization error recorded at toll gate. (**c**) Localization error recorded under the tunnel

For localization in the global reference frame, both the GPS and LiDAR PCM matching have been used. Figure 9a shows the pose accuracy of driving at K-City with GPS only. In all areas, except the toll gate and tunnel, GPS showed a very accurate position with less than 10 cm pose error. However, in the GPS-denied regions such as the toll gate and tunnel, the recorded pose error is 1.5 m to 8.5 m approx. Thus, GPS-based localization is only used in the outdoor region. For the tunnel and toll gate regions, the localization is performed with LiDAR PCM matching which estimates the pose using the normal distribution transform (NDT) [34]. The covariance matrix is determined for error optimization. NDT matching is used to determine a matching score for the covariance matrix. Each generated covariance matrix is used to update the pose in Kalman Filter (KF). The pose estimation results obtained after the pose correction using UKF are shown in Figure 9b. Figure 10 illustrates the localization error that occurred with both the above-mentioned schemes. With the map-based localization, the error is below 0.5 m.

In the worst case, if pose estimation is not possible through GPS or PCM matching, the vehicle pose is estimated by odometry for a few seconds and an emergency stop is activated when the vehicle appears to be out of control.

## 6. Perception

**Figure 11:**
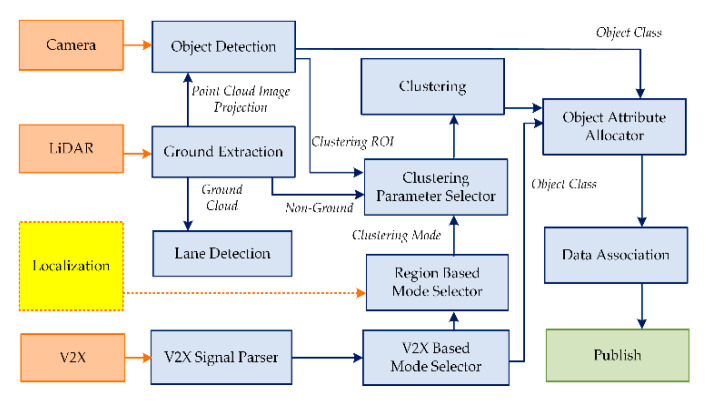
Perception structure overview.

The autonomous systems require a level of environmental perception that surpasses the human counterpart to fill the gap that arises due to the lack of prior knowledge and human instincts. A vast selection of off-the-shelf systems for vehicular automation is available for research and commercial use. However, a unified system capable of performing under all environmental conditions still remains the holy grail. The best road map in designing the perception system for an autonomous vehicle is to copy the behavior of the human counterpart. Driving in a dense urban environment requires focusing on numerous situations and objects (e.g., vehicles, pedestrians, pets, traffic lights, road zones, etc.), unlike in city outskirts, tunnels, and highways. Thus, the perceptual system for an autonomous vehicle needs a similar human trait to increase efficiency and to reduce the computational overhead. The perception module of Clothoid operates under a state machine, where each state modulates the parameters for perception corresponding to a specific environmental condition. The environmental perception of Clothoid relies on four sources of information: (a) LiDAR, (b) the camera, (c) GPS, and (d) the V2X communication module. Figure 11 illustrates the perception components. Each component of the perception is explained below. 

### 6.1. Ground Extraction

Clothoid performs object detection by using a camera and localizes the object in space through LiDAR. Thus, first of all, ground extraction is performed by separation of LiDAR point cloud measurements into the ground and non-ground point clouds. The technique deployed in this work follows a cylindrical grid-based approach with the non-planner ground assumption, as used in [35]. The point cloud related to ground measurements is used for lane detection in GPS-denied regions. The non-ground point cloud measurements are forwarded to object detection and clustering components.

### 6.2. Object Detection

The object detection component consists of a visual detector, you only look once (YOLO) version 3 [36], trained to recognize vehicles, pedestrians, and dynamic as well as static objects on the road. The visual detector localizes the objects in an image plane with a bounding box. To obtain a spatial location of an object, the LiDAR measurements are projected onto an image plane, and measurements corresponding to a specific box are processed. This component provides an approximation of the class and location of an object in the LiDAR frame, used for attribute allocation and clustering, respectively.

### 6.3. V2X Signal Parser

The V2X-enabled objects broadcast the relevant information that helps the autonomous vehicle in making decisions. The V2X information shared by the traffic signal indicates the location and the time remaining for the state change. Similarly, information of road blockage due to construction works on the road is communicated in terms of location. Moreover, V2X-enabled vehicles also share location and identity information. Primarily, identity, location and the state of V2X-enabled objects that exist beyond perceptual sensory enhance the overall decision-making process of autonomous vehicles. The V2X signal parser receives the information via the V2X modem and shares it with the relevant modules of the framework.

### 6.4. Region-Based Mode Selector

The region-based mode selector signals the mode flag to the clustering parameter selector, for the adjustment of the perception state. The clustering parameters are influenced by the location of the vehicle (through localization thread queried against the HD map) and the information shared by the V2X signal parser. The HD map identifies the environment to be a dense urban area, city outskirts, a highway, or a tunnel region. In contrast, information received over the V2X protocol enables the autonomous system to take appropriates decisions, such as giving way to approaching emergency vehicles; decisions regarding choosing alternate free paths; and preparing the vehicle to stop or pass the approaching traffic signal, considering the vehicle speed, distance to the signal and time remaining for signal change. The four basic mode flags are as follows:

1. General: The basic perception state that operates under standard roadway conditions where limited area ahead is scanned for all possible obstacles.
2. Dense urban: This flag increases the region of a scan with increased resolution, enabling the detection of pedestrians and other obstacles around.
3. Obstacle on highway: The categorization of a static or dynamic object on the highway ahead of the vehicle that needs to be treated as an obstacle.
4. Give way: As soon a fast-moving vehicle such as an emergency vehicle approaches from behind, the give way flag is turned on, and the vehicle has to change lane to clear the way for the approaching vehicle.

These mode flags are forwarded to the clustering parameter selector module for clustering of the LiDAR point cloud based on specific parameters.

### 6.5. Clustering Parameter Selector

This component preprocesses the received non-ground LiDAR point cloud based on the current perception state which is managed by the mode flags. The three main parameters altered in this module are the clustering region, the dimension filter, and the grid resolution.

Based on the clustering mode flag, the point cloud measurements are divided into a cartesian grid with specified resolution. Furthermore, the region of interest provided by the visual detector is enabled for LiDAR clustering, as the outlier count in visual detection is low.

### 6.6. Clustering

The clustering component makes the clusters of the LiDAR point cloud by following the connected component method [35]. The object clusters formed in the specified regions of the LiDAR point cloud are filtered through a dimension filter to further reduce the outliers. The LiDAR measurements are always partially occluded, so bounding box fitting through the L-shape fitting technique is applied along with a sparse occupancy array of points, representing center points occupied by clustering grid cells. The occupancy array of points is later used in local path planning.

### 6.7. Object Attribute Allocator

The centroids of filtered clusters corresponding to potential objects labeled by the LiDAR detector are examined by measuring the Euclidean distance [37] to the visually detected objects’ location (edge of objects). The distance value which is less than half of the width of the potential object gets the class of visual detector. Furthermore, attributes of dimensions are updated. All remaining objects carry the standard class of “obstacle” from the LiDAR detector to address the detection of unusual objects on the road.

### 6.8. Data Association

At the final stage of perception, all detected objects are traversed to merge the duplicates. The predicted class with the highest priority is assigned to the objects, and LiDAR-based occupancy arrays are used for spatial localization of objects. The detections are made in the vehicle frame, while the path planning and localization are carried out in the UTM coordinates. Thus, at the data association stage, all detected objects’ location and occupancy array points are transformed in the local UTM global coordinate system. The detected objects’ information can directly be used by path planning and control modules. The published information from the perception module includes the list of objects with dimensions, along with vehicle and UTM coordinate locations of object centroid and occupancy points. 

**Figure 12:**
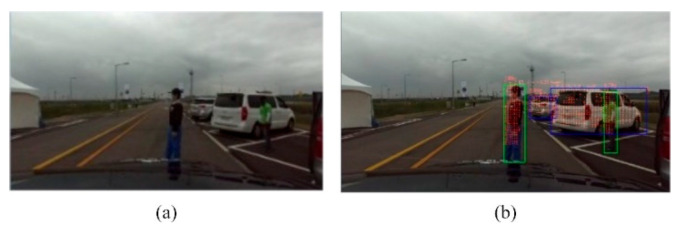
(**a**) Camera view. (**b**) Object detection through YOLO and LiDAR point cloud projection on the image plane for object spatial location.

For the evaluation of the perception module, Clothoid has been tested for the environment perception in K-City. Figure 12 illustrates the static and dynamic object detection in the real environment while the vehicle is driving in the dense urban region. The pedestrians and vehicles are localized with the bounding box, and the LiDAR point cloud is projected in the bounding box for the spatial location of each object. As soon the pedestrian is detected on the road, the perception module sends a flag to the control module to stop the vehicle until the pedestrian walks away from the lane.

## 7. Path Planning

The path planning module is responsible for global path planning and local path planning. The global path planner generates the cost-based shortest path on the map. Using this global path information, the local path planner generates a smooth trajectory for the vehicle. Each of these is explained in the below sections.

### 7.1. Global Path Planning

One of the major contributions of this research is the development of a global path planner that not only finds the cost-based optimal global path but that is also robust to dynamic changes in the environment. The generated global path strictly obeys the driving rules in the urban region and highway road. The steps involved in global path planning are explained below.

#### 7.1.1. Topological Graph Generation

**Figure 13:**
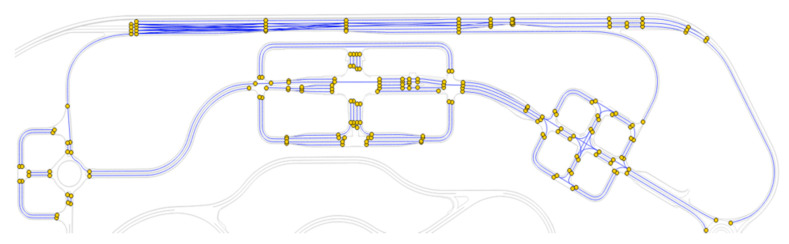
The generated topological graph using the HD map.

After the HD map development, the global path planner firstly generates a topological graph for the complete map which includes urban and highway regions, as shown in Figure 13. The generated topological graph is the connected graph. To generate a connection between the start and the end point along the complete path, the two end points of each edge were collected as the nodes. The Euclidean distance, computed by the HD Mapping module, of each edge was used for finding the neighboring nodes. 

#### 7.1.2. Optimal Path Search

For navigation of an autonomous vehicle, it is necessary to generate an optimal global path among the available routes. Once the global path is generated, the local path planner calculates the optimal trajectory for that path. Depending on the map analysis, different approaches can be used for global path planning such as roadmap-based path planning, i.e., Voronoi [38], Dijkstra [39], Best First [40], A* [41], map decomposition into regions or cells [42], random trees [43], and neural networks [44]. 

**Figure 14:**
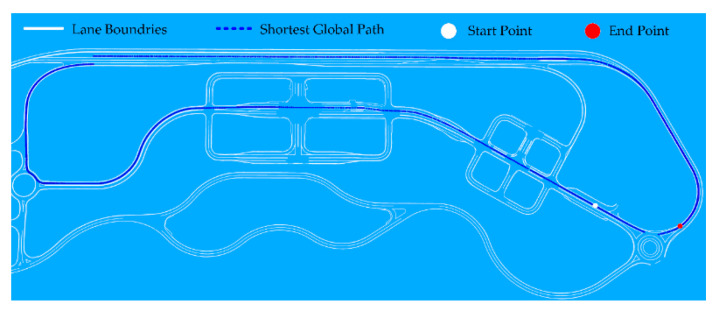
Global shortest path from the start point to the end point.

In Clothoid, the complete map is already generated by the HD mapping module, where the complete route network is available. Thus, we implemented the Dijkstra algorithm which is a greedy algorithm for finding the shortest path between the source and destination nodes. The algorithm finds shortest paths from the source to all other nodes in the graph, producing a shortest-path tree which is helpful when the path between any two neighboring nodes on the graph is blocked due to traffic congestion. If any sub-path on the global path is blocked, the Dijkstra algorithm, instead of finding a complete path from the source node to the destination node, finds an alternative sub-path on the shortest path tree and publishes the new global optimal path from the current position of vehicle to the destination node. The shortest path tree is computed by finding the neighboring nodes at each instant. Two methods can be used for finding the neighboring nodes in the route network: the adjacency matrix and adjacency list [45]. The adjacency matrix is useful in the case of dense graphs. As the topological graph generated for K-City is sparse, and the maximum connections for any node are no more than four, the adjacency list is used due to high suitability for sparse graphs. Figure 14 illustrates the shortest path, generated from the start point to the end point using the Dijkstra algorithm. The shortest global path waypoints lie at the center of the lane, thus enabling the vehicle to drive in the center of the lane while maintaining the safety margins at all times. 

#### 7.1.3. V2X-Based Path Planning

The traditional approaches used for global path planning include:

1. Generating the path for the pre-known map where the static obstacles’ information is already available and the global path is generated by avoiding those static obstacles. Such a method is not feasible for dynamic environments.
2. Generating the path heuristically when the complete map is not pre-known. In this approach, an autonomous vehicle usually perceives the environment using the camera and LiDAR, which enables the vehicle to detect the obstacles in a limited field of view. Using this information, the path is generated by avoiding these obstacles.

**Figure 15:**
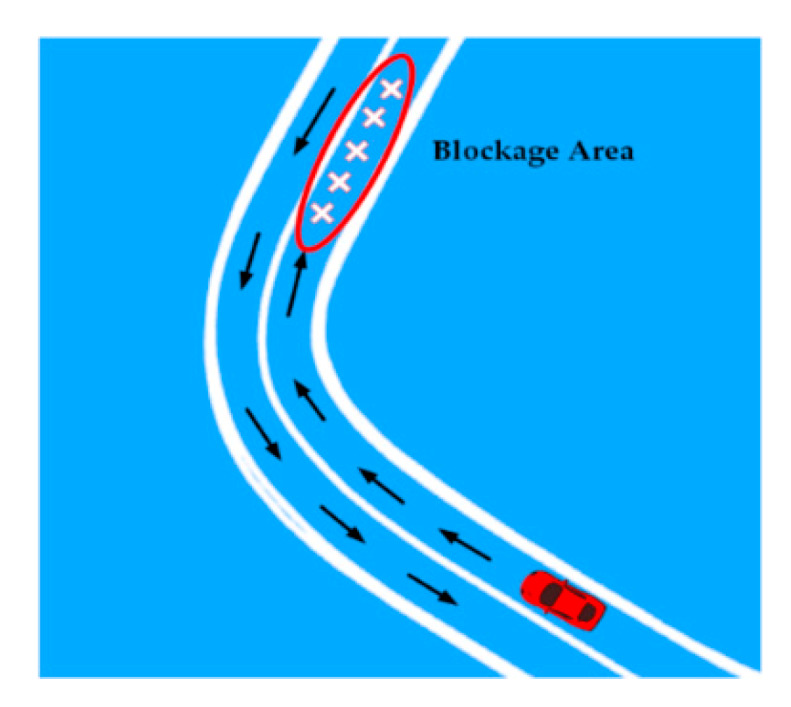
Temporary road blockage on the global path.

The above-mentioned approaches fail in the scenario when the vehicle is following a certain global path and a blockage dynamically occurs on the path due to unfavorable situations such as road accidents, heavy traffic jams, or road construction, as shown in Figure 15.

**Figure 16:**
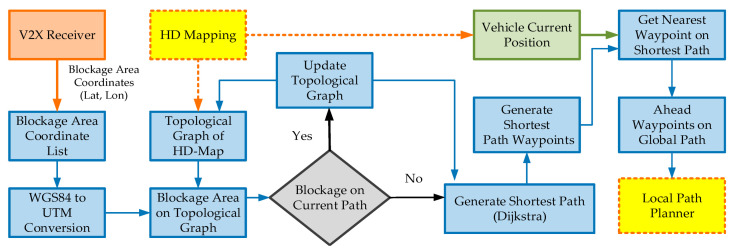
Vehicle-to-everything (V2X)-enabled global path planning model.

An autonomous vehicle must be able to avoid such situations. This problem can be solved by the V2X communication where the vehicle can receive the blockage information from the infrastructure. In recent years, vehicle-to-vehicle (V2V) and vehicle-to-everything (V2X) communication have received great attention from autonomous driving industries. Due to the benefits of 5G-V2X technology, many researchers have proposed path planning techniques for autonomous vehicles. Shi et al. [46], for example, proposed a heuristic motion planning algorithm for safety motion planning at the intersections with the usage of V2V communication. Similarly, Hussein [47] studied the importance of V2X communication in off-road environments. The Clothoid framework develops a novel V2X-based global path planning module which enables the vehicle to robustly change the path in the dynamic environment. Figure 16 illustrates the working model of the novel V2X-enabled global path planner of the proposed Clothoid framework. 

The V2X transmitters installed in the road infrastructure broadcasts the information about road blockage to all the autonomous vehicles in their transmission range. The V2X receiver, embedded in the vehicle, can receive the V2X messages within a range of 200 m. The received V2X messages, that is, the UDP packets of the traveler information message (TIM), contain the blockage area location coordinates. The V2X-enabled path planner extracts this information and performs the following operations:

1. It maintains a list of blockage areas through the V2X message using Algorithm 1.
2. It converts the blockage area location coordinates from WGS84 into UTM coordinates.
3. It localizes the blockage area in the topological graph generated from the HD map using Algorithm 2.
4. If the blockage area is on the pre-generated global path, within the range of 200 m, it updates the topological graph and regenerates the shortest global path among the alternative routes by applying Algorithm 3. This property allows the autonomous vehicle to avoid the unfavorable situations occurring due to the dynamic changes along the path.
5. The V2X transmitter repeatedly forwards the road blockage V2X message at a frequency of 10 Hz. If there is any new blockage area information in the V2X message, the global path planner immediately updates the blockage area list in Algorithm 1 and repeats the above-mentioned operations.
6. It generates the waypoints of the complete shortest global path computed by the Dijkstra algorithm.
7. At each timestamp, the global path planner uses the vehicle’s current position and groups the waypoints ahead on the global path that are local to the vehicle pose. These waypoints are forwarded to the local path planner.

**Figure 17:**
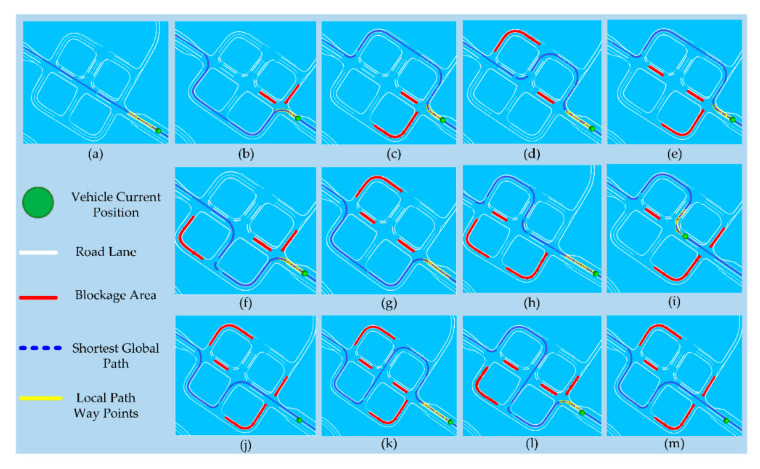
Ground test scenarios for road blockage. (**a**) No road blockage, (**b**–**m**) different road sections blocked.

**Algorithm 1:** Collecting blockage region information from the V2X receiver**Input:** V2X message **Output**: List of blockage regions’ coordinates Index = 0; **If**
*V2Xmessage list is not empty*
**then**                start_point_coordinates [0] = V2X_message_blockage_area [0];                end_point_coordinates [0] = V2X_message_blockage_area [1];                **If**
*start_point_coordiantes*
**not in**
*Blockage_region_list*
**then**                               Blockage_region_list [index]. append (start_point_coordiantes);                              Blockage_region_list [index]. append (end_point_coordiantes);                               Index++; Visualize Blockage regions on HD Map; **Return** Blockage_region_list;

**Algorithm 2:** Detect road blockage regions on topological graph**Input:** List of UTM coordinates for blockage regions, topological graph **Output:** List of graph edges corresponding to blockage regions. Index = 0; **for**
*Blockage_region_list*
**do**,                Shortest_distance = 0;                **for**
*each blockage region in Blockage_region_list*
**do**                X = start_point_x in start_point_coordiantes; Y = start_point_y in start_point_coordiantes; **for**
*all the edges in topological graph*
**do** Compute Euclidean distance from X,Y to waypoints of edges; **If**
*Euclidean distance < Shortest_distance*
**then** closest_edge = waypoint with shortest Euclidean distance;         If *closest_edge is not in Blockage_Graph_Edges_List*
**then**                              Blockage_Graph_Edges_List[index].append(closest_edge);                              Index++; **Return** Blockage_Graph_Edges_List;

**Algorithm 3:** V2X-based global path update**Input:** Shortest_global_path from start point to end point, Blockage_Graph_Edges_List **Output:** Updated shortest_global_path **for**
*edges in Blockage_Graph_Edges_List*
**do**          **If**
*edge in shortest_global_path*
**then**                   graph.remove(edge); shortest_global_path = Generate_shortest_path_using_dijkstra(graph); Visualize shortest_global_path on HD Map; **Return** shortest_global_path;

For the evaluation of the V2X-enabled global path planner on the proving ground, the V2X receiver was calibrated in the vehicle and programmed to detect the blockage area in the range of 200 m. The roadside units (RSUs) were set up by the Hyundai [48] across the road which broadcasts the blockage area information to the vehicles as a TIM. The road blockage data were collected by running the vehicle in K-City multiple times. The experiments were performed for 12 different road blockage scenarios and a no-blockage scenario. The V2X-based global path planning results are visualized using Rviz as shown in Figure 17. Figure 17a shows the global path for no blockage scenario, and Figure 17b–m illustrates the results for blockage scenarios where the global path has been dynamically generated according to the blockage information received from the V2X module. The results show that the V2X-enabled path planner can robustly regenerate the new path within the range of 200 m, thus avoiding the blockage region beyond the line of sight and providing unlimited environmental perception. 

### 7.2. Local Path Planning

The optimal global path generated by the global path planner may consist of different zones with different speed limits, such as an urban zone consisting of a school region, where the roads are more congested and are steeper, or a highway zone where the vehicle needs to control the speed accordingly. Clothoid’s local path planner is designed to control the vehicle in such situations. Thus, after receiving the shortest path from the global path planning module, the local path planner computes an optimal trajectory for each zone based on the road structure and speed limit.

The major operations of Clothoid’s local path planner include the smooth trajectory generation, lane change with jerk minimization, and obstacle avoidance.

#### 7.2.1. Smooth Trajectory Generation

The local path planner receives the ahead waypoints from the global path planner as an input.

These waypoints are in UTM coordinate system and are not equidistant to be used for a local planner. To generate the smooth trajectory along the given path, the local path planner converts the global waypoints into the vehicle’s local coordinates and then applies the N-order polynomial function to convert the local waypoints into the equidistant smooth trajectory. For the estimation of polynomial function coefficients, the QR factorization is applied [49].

#### 7.2.2. Lane Change with Jerk Minimization

Among the other autonomous functions, one of the most exciting abilities of Clothoid is that it is designed to follow the traffic rules. Throughout the path in the urban region, the vehicle always stays in the right lane, follows the traffic signs, moves to the high priority lane on the highway, and empties the lane on the arrival of an emergency vehicle, which is one of the social-friendly features of Clothoid. Thus, as soon the vehicle enters the highway road, the highest priority lane should be followed. For this purpose, a vehicle must change lane and reach the highest priority lane, provided that it is free. While driving on the lane, Clothoid continuously observes the other vehicles, and if there is an emergency vehicle approaching from behind, the vehicle changes lane to make the lane free. After the emergency vehicle passes, the vehicle changes lane and moves back to the high-priority lane.

**Figure 18:**
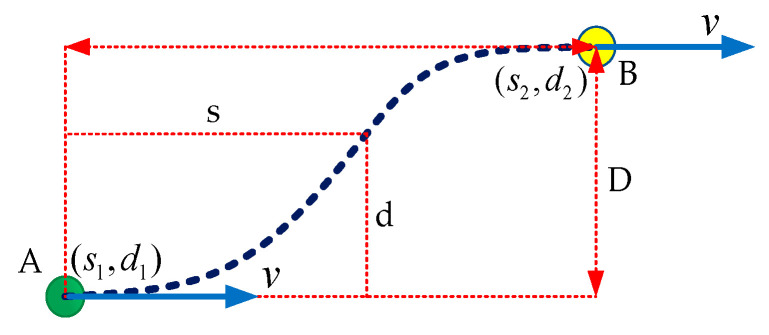
Longitudinal and lateral trajectory generation in the Frenet frame.

During the lane change operation, the local path planner must generate the trajectory from point A, ahead of the vehicle, on the current lane to point B on the destination lane, acting as the start and end points, as shown in Figure 18.

Along the generated trajectory, the jerks need to be minimized to keep the trajectory smooth. In this research, the Frenet frame [50,51] is used, referenced to the centerline of each lane to combine lateral and longitudinal motion. In Figure 18, the parameters *s* and *d* represent the longitudinal distance and lateral displacement to the initial temporal position A. To mimic the human-like driving behavior, the lateral and longitudinal trajectory is generated as a function of time using quantic fifth-order polynomial functions:

(2) $$f_{s}(t)=\sum_{i=}^{5}\alpha_{i}t^{i}$$

(3) $$f_{d}(t)=\sum_{i=}^{5}\beta_{i}t^{i}$$

where $\alpha_{i}$ and $\beta_{i}$ are the coefficients of the functions $f_{s}$ fsand $f_{d}$.

The above polynomial functions are the jerk-optimal connections between the start point A and end point B. As in our case, the lane change event occurs at the highway, it is assumed that the velocities $v_{1}$, $v_{2}$ and heading angle before the lane change at point A, and after the lane change at point B, remain the same. Furthermore, the values of acceleration $a_{1}$, $a_{2}$ are assumed to be zero at both states. The velocity v, acceleration a, and jerk j are computed by taking the derivates of the above polynomial functions at each time step T = 0.1 to generate a jerk-free trajectory given as:

(4) $$v=\frac{df}{dt}$$

(5) $$a=\frac{d^{2}f}{dt^{2}}$$

(6) $$j=\frac{d^{3}f}{dt^{3}}$$

Through experiments, the optimal values of velocity were chosen to generate the optimal travel time, given as:

v[10→5]ms→[1.5→2.5]s

The above values are used to generate a smooth trajectory along the path during the lane change.

#### 7.2.3. Obstacle Avoidance

**Figure 19:**
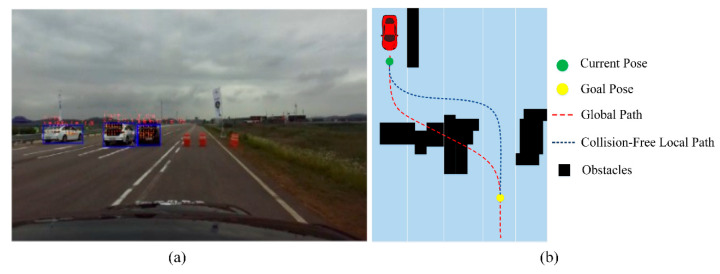
(**a**) Obstacles detected by the perception module, (**b**) obstacle avoidance and local path generation on the occupancy grid map.

Another important function for safe driving of an autonomous vehicle is to drive on the road by avoiding obstacles such as road blockers or any static vehicles on the highway road due to accidents or technical faults. The perception module detects these obstacles, as shown in Figure 19a, and identifies the spatial location of each obstacle ahead of the vehicle on the global HD map. If the obstacles are lying on the predefined global path and the generated local trajectory, the obstacle avoidance mechanism is activated. In this case, the local path planner must detour the global path and is responsible to generate a new path while taking the non-holonomic constraints into account to avoid a collision with the obstacle. Clothoid’s local path planner heuristically searches for the obstacle-free path on the global map by using a similar method as implemented in [52,53]. Initially, it takes the vehicle’s current pose at any nearest point ahead of the vehicle, goal pose, and the occupancy grid map as the input. The size of occupancy grid map provided to the algorithm is a 30 m range window along the global path, starting from the initial pose. As the position of the obstacle is provided by the perception unit, the occupancy grid map is populated and consistently updated through a sliding window approach with the obstacle information to generate the optimal path in real time ~10 Hz. Based on the grid map and global path, the goal pose is estimated for the vehicle which is required to be achieved after the obstacle avoidance. Using the current and goal pose, the locally optimal path is searched on the available free cells of the grid map through a hybrid A* algorithm [52,53], as shown in Figure 19b. The holonomic constraints for the generated path given in the obstacle distance lookup table and collision lookup table are used to generate the collision-free path. In addition, the resolution of occupancy grid map is set to 0.5 m, and the cell, after being labeled as occupied, requires five consecutive time steps of being vacant to be considered as traversable. This check is set in place to address the missed detections and to ensure the collision-free path. This path is fed to the smoothing unit which optimizes the generated path to enhance the distance from the obstacles such that the collision risk is minimized, making the trajectory smooth while passing through the obstacles. The resulting collision-free local path is forwarded to the control module. To enhance the path generation rate and make the path planner robust in the presence of dynamic obstacles, the path is regenerated for a smaller region around the detected obstacle instead of fixing the goal pose in the larger area. This distinguishing feature makes Clothoid able to regenerate the optimal path multiple times for multiple detected obstacles even at very short distance values.

## 8. Control

**Figure 20:**
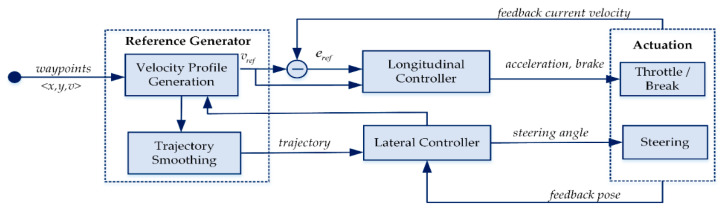
The control unit of Clothoid.

The role of a controller is to steer the vehicle on the given path under the safety constraints with the reference velocity. The control component of Clothoid, as shown in Figure 20, receives a local trajectory from the local path planner which consists of the specified range ahead of the waypoints and maximum velocity limit. It consists of two major components: the reference generator and the controllers.

### 8.1. Reference Generator

The reference generator is responsible for the velocity profiling of the input trajectory based on the perceived environment. At each time step, the maximum velocity limit is specified for the future path of the vehicle. As per the K-City rule, the speed is limited to 40 km/h and 80 km/h in the urban environment and highway road. The velocity profiling module generates an accurate velocity for the given trajectory. The generated velocity associated with each waypoint of the trajectory is dependent on the structure of the road, i.e., the straight lane or the curve, the obstacles on the road, the traffic signs, etc. The resulting trajectory is forwarded to the controller which consists of the longitudinal and lateral control components.

### 8.2. Longitudinal Controller

The longitudinal controller takes the reference velocity $v_{ref}$ as an input and applies the feed-forward Proportional Integral (PI) control method [54,55] which tracks the feedback signal containing velocity error $e_{ref}$, windup effect before and after saturation $e_{se}$, and the vehicle velocity $v_{FPI}$ is yielded as:

(7) $$v_{FPI}(t)=K_{FF}v_{ref}+K_{p}e_{ref}+(K_{I}+K_{AW}e_{se})\int_{t=t_{1}}^{t_{2}}e_{ref}(t)dt$$

$K_{FF}(v)$, $K_{p}(v)$ and $K_{I}(v)$ represents the feed-forward, propagation, and integration coefficients, respectively.

**Figure 21:**
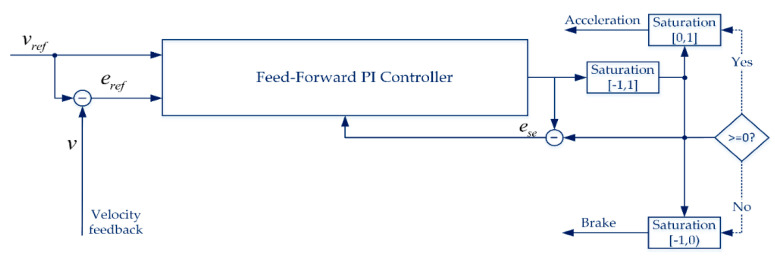
Feed-forward PI controller.

$e_{se}$ limits the acceleration and brake, which are fed into the throttle and brake of the vehicle base model, between −1 and 1. The longitudinal controller controls the vehicle speed by minimizing the error $e_{ref}$ between the reference and current velocity. Figure 21 illustrates the working model of the feed-forward PI controller.

### 8.3. Lateral Controller

**Figure 22:**
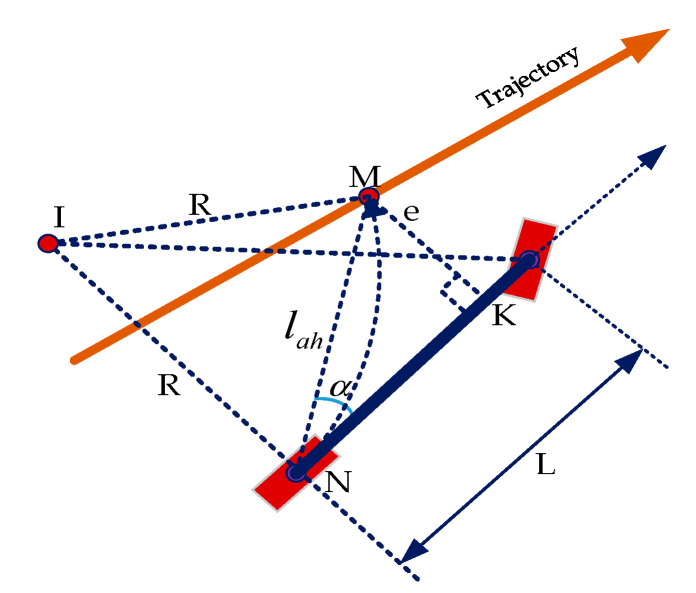
The steering angle estimation using pure pursuit control.

The lateral controller is responsible for tracking the path being followed by the vehicle and maneuvering the steering angle to ensure that the vehicle continues to follow the input trajectory. In this research, the pure pursuit control method [54,56] is applied due to the stabilization against disturbances [57] for the lateral controller instead of deep learning methods [58,59,60] which are more complex, computationally expensive and less robust to the real environment. Figure 22 illustrates the steering angle computation by using the pure pursuit control method. At each time step, the steering angle *θ* towards the ahead waypoint M lying on the circle of radius R with center point I and a tangent *e* at M is calculated as:

(8) $$\theta =\tan^{-1}\left(\frac{2L\sin\alpha }{l_{ah}}\right)$$

where

L = the distance between the left-front and left-rear wheel of the vehicle, i.e., K and N, specified in meters;
α = the angle between vehicle heading and direction of M;
$l_{ah}$ = the look-ahead distance.
$l_{ah}$ is computed as a function of velocity given as:

(9) $$l_{ah}(v)=\left\{\begin{array}{cc} \begin{array}{c} 2\\ 0.45v-2.5\\ 20 \end{array} & \begin{array}{c} v<10\mathrm{km}/h\\ 10\mathrm{km}/h\leq v\leq 50\mathrm{km}/h\\ v>50\mathrm{km}/h \end{array} \end{array}\right\}$$

The actuation is performed based on the resulting steering angle and velocity.

**Figure 23:**
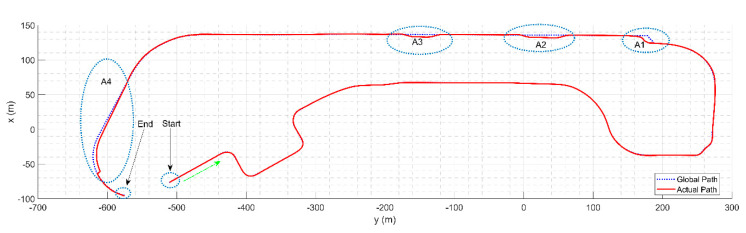
Path tracking results from the start to the end point.

Figure 23 shows the shortest global path generated for the road blockage scenario in Figure 17c. The results for path tracking through the whole trajectory can be analyzed, where A1, A2, A3 and A4 represent the regions with dynamic obstacles, as shown in Figure 19, lane change for emergency vehicles and the GPS-denied region i.e., the tunnel. The results indicate that the controller is able to robustly handle the vehicle motion with low slip when compared to the reference global path.

**Figure 24:**
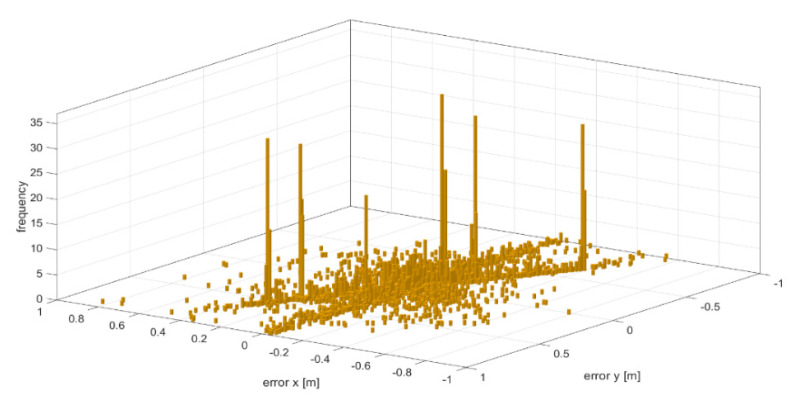
Path tracking error in X and Y directions.

The path tracking error along the path is shown in Figure 24. Along the straight path, the average tracking error per waypoint is 6 mm, while it rises to 25 mm in the GPS-denied regions and regions with high curvature, i.e., A1, A2, A3 and A4.

In the GPS-denied regions, the tunnel and toll gate, the localization and the path tracking error can be more extensive. However, in our case, in spite of the localization error, which is very minor for such a region, the controller works and follows the generated path smoothly. This is achieved by the filtering out the localization error at each time step. The state estimation provides an exceptionally smooth base on UKF localization, which handles the probabilistic theorem to filter out the error. The information for control is smooth and does not affect the controller, resulting in a jerk free navigation.

## 9. Conclusions

In this research, a unified software framework for fully autonomous vehicles named Clothoid has been presented. The proposed framework is enabled to handle the challenging conditions in real environments including urban areas and highway. Clothoid integrates all autonomous driving modules, such as perception, localization, planning and control into a hierarchical structure which allows the rapid development and easy testing of the proposed framework. The distinguishing features of Clothoid include the ability to handle challenging situations such as detect and avoid pedestrians, evade construction sites, avoid static and dynamic obstacles, make way for ambulances, and localize the vehicle in GPS-denied regions. Furthermore, a novel V2X-enabled global path planner has been developed and integrated with Clothoid to ensure long-range route planning while avoiding dynamic environmental conditions such as road blockages due to traffic congestion or road construction. The performance of each module has been validated in K-City under multiple scenarios. This research proves that all modules of the proposed unified framework operate together to successfully drive the vehicle from the starting position to the goal point in a dynamic environment without any collisions. We aim to extend the scope of our work by addressing the severe dynamic situations on the road, such as avoiding cheating vehicles and bicycles on the road, driving in the off-road environment and ensuring the robustness in highly dynamic environmental conditions such as weather changes.

## Figures and Tables

**Table 1:** Reference frames and priorities for each method used in unscented Kalman filter (UKF)-based localization.

Methods	UKF Stages	Reference Frame Axes	Priority
IMU odometry	Prediction	Local	4
GPS	Correction	Global	1
LiDAR odometry	Correction	Local	3
LiDAR map matching	Correction	Global	2
